# Applying Deep Neural Networks and Ensemble Machine Learning Methods to Forecast Airborne *Ambrosia* Pollen

**DOI:** 10.3390/ijerph16111992

**Published:** 2019-06-04

**Authors:** Gebreab K. Zewdie, David J. Lary, Estelle Levetin, Gemechu F. Garuma

**Affiliations:** 1 William B. Hanson Center for Space Sciences, The University of Texas at Dallas, Richardson, TX 75080, USA; david.lary@utdallas.edu; 2 Department of Biological Science, The University of Tulsa, Tulsa, OK 74104, USA; estelle-levetin@utulsa.edu; 3 Institute of Earth and Environmental Sciences, University of Quebec at Montreal, Montreal, QC H2L 2C4, Canada; gemechufanta@gmail.com

**Keywords:** *Ambrosia* pollen, random forest, extreme gradient boosting, deep neural networks, machine learning, ECMWF, pollen allergy

## Abstract

Allergies to airborne pollen are a significant issue affecting millions of Americans. Consequently, accurately predicting the daily concentration of airborne pollen is of significant public benefit in providing timely alerts. This study presents a method for the robust estimation of the concentration of airborne *Ambrosia* pollen using a suite of machine learning approaches including deep learning and ensemble learners. Each of these machine learning approaches utilize data from the European Centre for Medium-Range Weather Forecasts (ECMWF) atmospheric weather and land surface reanalysis. The machine learning approaches used for developing a suite of empirical models are deep neural networks, extreme gradient boosting, random forests and Bayesian ridge regression methods for developing our predictive model. The training data included twenty-four years of daily pollen concentration measurements together with ECMWF weather and land surface reanalysis data from 1987 to 2011 is used to develop the machine learning predictive models. The last six years of the dataset from 2012 to 2017 is used to independently test the performance of the machine learning models. The correlation coefficients between the estimated and actual pollen abundance for the independent validation datasets for the deep neural networks, random forest, extreme gradient boosting and Bayesian ridge were 0.82, 0.81, 0.81 and 0.75 respectively, showing that machine learning can be used to effectively forecast the concentrations of airborne pollen.

## 1. Introduction

Pollen allergies are prevalent in the temperate regions of the northern hemisphere. The USA, southern Canada and south–central Europe all have significant populations experiencing pollen allergies [1,2]. In North America alone, about 50 million people have allergic reaction to pollen in the form of asthma, hey fever, and allergic rhinitis [3,4]. In Europe, as of 2007, the prevalence of pollen allergies is nearly 40% of the total population [2] and the sensitization is expected to double, at least for ragweed pollen, over the period 2014–2060 [5]. The effect of pollen is not well studied in the developing world and it is often masked by the effect of dust in tropical regions [6].

Weed plants particularly the *Ambrosia* species, e.g., *Ambrosia artemisiifolia* (common ragweed), *Ambrosia trifida* (giant ragweed) are the major producers of large amounts of pollen and are known in their allergic potency. For example, common ragweed can produce up to about 2.5 billion pollen grains per plant per day [7]. *A. artemisiifolia* and *A. trifida* together can produce more allergens than all other plants combined [8]. Following *A. artemisiifolia*, Grasses (e.g., rye grass) can trigger strong allergic response [8,9]. Pollen produced by trees can also cause an allergic response that is typically less than that of weeds and grasses. In some particular regions tree pollen can trigger a significant allergic response. For example, the airborne concentration of mountain cedar pollen grains can reach tens of thousand of pollen grains per cubic meter and trigger a significant allergic response in central Texas during winter, known as cedar fever [10,11].

In addition to the strong association between the airborne pollen concentration and the incidence of asthma and other allergic reactions, previous studies have shown that high-levels of airborne pollen are also associated with other public health issues. For example, Low et al. [12] have shown the association between high pollen abundance and hospital admissions to stroke. The link between female suicide mortality and the atmospheric pollen concentration in Tokyo, Japan is present by Stickley et al. [13]. Additionally, Hanigan and Johnston [14] have shown the association between high pollen abundance and hospital admissions of both asthma and Chronic Obstructive Pulmonary disease (COPD).

The distribution of pollen in the atmosphere and its allergic effect show a significant variation in both rural and urban areas. This variation is associated with suitability of that area for growing the weeds, grasses, or trees producing the pollen and the ambient weather and climate. Generally, pollen abundance is higher in rural areas than cities and urban areas. Contrarily, pollinosis (pollen caused allergy) is higher in urban than rural environments [15]. This can be associated with the difference in thermal, hydrological, precipitation and concentrations of pollutants between urban and rural areas [16,17,18].

The amount of pollen in the atmosphere depends on many factors, such as the vegetation type and coverage distribution in the area, climate and weather, the prevailing wind, geographical conditions, and so on [19,20]. Estimation and forecasting of allergic pollen has been done based on these factors using linear and nonlinear regression techniques, advanced computational machine learning methods such as the multi-layer perception (MLP) neural network [3,21] and ensemble based machine learning methods, such as random forests [1].

In addition to their recent application to estimate pollen, machine learning methods have been popular to study particulates having serious environmental health problems. Environmentally hazardous particulates such as PM${}_{2.5}$, PM${}_{10}$, NO${}_{2}$, CO, Ozone are estimated using different machine learning methods [22,23,24]. A few studies, particularly made in the European region, have employed machine learning methods to estimate pollen abundance. For instance, Castellano-Méndez et al. [25] and Puc [26] have used the simple neural network to predict allergic *Betula* pollen over Spain and Poland, respectively. Csépe et al. [21] used different Computational Intelligence (CI) methods to predict the *Ambrosia* pollen at two different places in Hungary and France. Recently, machine learning methods were applied to predict allergic pollen over the United States. For example, Refs. [27,28,29,30] used different machine learning methods to estimate allergic *Ambrosia* pollen at Tulsa, Oklahoma.

Pollen measurements made at a certain area are occasionally transported from other locations [31,32,33]. Investigation of air mass movement over that location prior to the pollen observation time is necessary to characterize airborne pollen movement. Previous studies show that large pollen observations and abrupt day to day variations over a certain location are associated with directional air mass transport [34,35].

Most of pollen forecasting techniques, especially those made over the European region, stress the importance of meteorologic variables [36] in forecasting allergic pollen. Recently, Zewdie et al. [30] used the NEXRAD weather radar measurements to estimate *Ambrosia* pollen. And only few studies employed advanced machine learning methods.

The objective of this study is therefore to forecast the airborne *Ambrosia* pollen abundance using advanced machine learning methods such as deep neural networks, random forests and extreme gradient boosting ensemble learners. For comparison, we also used the linear Bayesian ridge machine learning method. The environmental context data used is drawn entirely from the European Centre for Medium-Range Weather Forecasts (ECMWF) atmospheric weather and land surface reanalysis datasets. Table 1 shows the ECMWF parameters utilized in our predictions.

## 2. Materials and Methods

### 2.1. Pollen Samples

**Figure 1:**
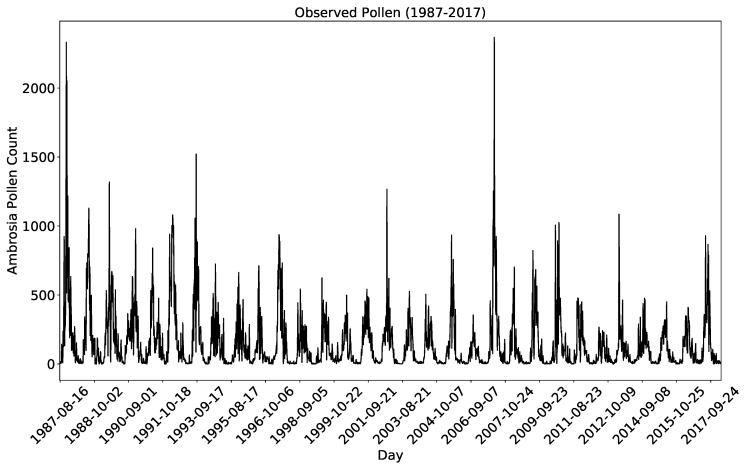
Showing the actual *Ambrosia* pollen used to supervise the machine learning models. The first set of data from 1987 to 2011 is used to develop all the machine learning models. The rest data from 2012 to 2017 is used to test the prediction performance of the models.

Pollen sampling was performed at the University of Tulsa, Oklahoma (36.1511° N, 95.9446° W). Preliminary analysis of the pollen data measured from 1987–2014 and a detailed description of the technique and study area is given by [3]. Figure 1 shows a time series plot of the daily pollen concentration measurements made from 1987 to 2017 during the *Ambrosia* pollen season (15 August–31 October).

A volumetric spore-trap apparatus of the Hirst type [37] was used to collect the *Ambrosia* pollen from the top of a 12 m high building inside the University. The apparatus works by directing air through a tiny orifice using an external vacuum pump. Inside the apparatus, directly below the small orifice, there is a slowly rotating clockwork drum (rotating at a velocity of 2 mm per hour) covered in a greased tape. The pollen data collected in this way [37] is shown in Figure 1 and was used for the supervised machine learning methods.

### 2.2. ECMWF Data Description

ECMWF reanalysis, Ref. [38] data was used as a source of the daily meteorological and land surface contextual data. The predictor variables utilized include the total water column, cloud cover, surface and mean sea level pressures, vertical and horizontal wind speed, soil temperature at various levels, skin temperature, surface albedo, total column ozone, volumetric soil water, dew point temperature at 2 m, surface and 2 m temperature and precipitation, high and low vegetation cover, etc. Table 1 shows the list of our predictor variables and their units.

While most of the names of these variables are self explanatory, some of the variables need a brief description. For example, the skin temperature is the temperature of the surface layer of the Earth. It is different from the meteorological definition of surface temperature which refers to the temperature measured by a thermometer placed at approximately 1 m above the ground. The skin temperature shows a higher diurnal variation than temperatures measured above the ground. The total column ozone refers to the total amount of ozone in a vertical column from the surface of the earth to the top of the atmosphere (kg m${}^{-2}$). The ECMWF soil temperatures at level 1, 2, 3 and 4 refer to the average temperature at layers, respectively, from 0–7 cm, from 7–28 cm, from 28 cm–1 m, and from 1–2.89 m deep. The skin depth reservoir (measured in mm) is another land surface parameter referring to the the amount of water accumulated in interception reservoirs. The surface albedo is a measure of the surface reflectivity. The surface albedo of the earth is the fraction of the incident sunlight reflected [39].

### 2.3. Machine Learning Methods

Machine learning is a powerful approach to build empirical models from data alone. They are particularly useful for problems in which the relation between parameters is complicated and we do not know a priori the functional relationship between the input and output parameters [27]. For machine learning methods to effectively estimate an environmental variable we need to provide as a comprehensive set of variables comprising as many variables as possible. Nowadays machine learning methods are gaining widespread application in different areas ranging from radio astronomy to atmospheric research, from identification of spam emails to detection of online fraud [27,40,41].

### 2.4. Procedure

The Hirst, Ref. [37], pollen trap apparatus was used to measure the daily pollen concentration from 1987 to 2017. Results of a multiple regression model and description of the data from 1987 to 2013 was given by [3]. For this research, the pollen data measured form 1987 to 2011 was used to develop the machine learning models and the last six years of data from 2012 to 2017 was used to test the prediction performance of the machine learning models. Since the range of the data values varied widely for different predictor parameters, normalization of the each parameter was essential. Some machine learning algorithms, for example deep neural networks, may not produce correct results if all the input parameters are not normalized. In this research, each input parameter was normalized by subtracting the mean and dividing by the standard deviation of the parameter to implement the deep neural network algorithm. The random forest, extreme gradient boosting, and Bayesian ridge algorithms worked well independently of normalization. In order to identify the relationship between the pollen abundance and previous days atmospheric weather and land surface parameters, all input parameters were time lagged up to 30 days back.

The deep neural network models were developed with two hidden layers each with 64 neurons and the model trained for 500 epochs. The keras [42] library from python was used to implement the deep neural network and is run on top of tensorflow [43].

The random forest and extreme gradient boosting machine learning methods were trained with 1000 trees each. Five-fold cross validation was used to train the extreme gradient boosting machine learning method. The Bayesian ridge machine learning model was trained for 3000 iterations. The Pearson correlation coefficient is used to evaluate the performance of all machine learning methods.

In order to see the relationship between the present day pollen and previous days atmospheric weather and land surface parameters each predictor parameter was lagged by one to 30 days. The total training data was then the product of the number of parameters in Table 1 and 30.

In order to characterize high day to day pollen fluctuations back trajectory analysis was performed for a selected number of days in 2010. In this case we plot the path of the movement of a parcel of air in a short period of time [34]. We used the HYSPLIT back-trajectory [44] analysis and the trajectories were run for every four hours six times a day at an altitude of 12 m at the location of the pollen collection site.

## 3. Results

**Figure 2:**
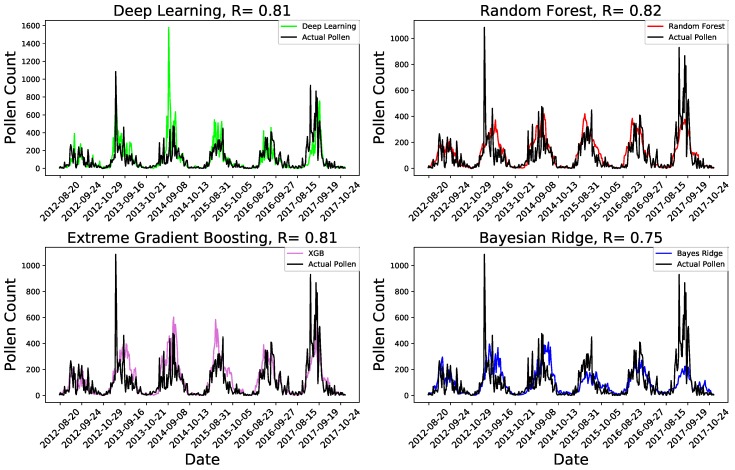
The predicted *Ambrosia* pollen using deep learning, random forest, extreme gradient boosting and Bayesian ridge machine learning methods for the pollen seasons from 2012–2017. The black curve in all subplots depicts the actual pollen for the same period. The colored lines show the predicted pollen using an independent dataset from 2012–2017. In each case the machine learning model was developed using data from 1987–2011.

Figure 2 shows the pollen abundance estimated using the four machine learning methods (colored lines) and the actual pollen abundance (black line) for 6 years from 2012 to 2017. The black curve in all subplots shows the actual pollen withheld for independent testing. The colored plots show the estimated pollen abundance using the independent test dataset. The type of machine learning method used and Pearson correlation between the forecasted and the actual pollen are given at the top of each figure for all subplots. The Pearson correlation values for deep learning, the random forest, extreme gradient boosting and Bayesian ridge were, respectively, 0.81, 0.82, 0.81 and 0.75.

From Figure 2, we observe that the different machine learning methods and deep learning predicted the pollen abundance with correlation coefficient values indicating superior performance. The deep neural network, the random forest and extreme gradient boosting methods produced comparable results. The random forest machine learning method produced the most robust prediction with a correlation value of 0.82. Relatively, the poorest performance is exhibited by the linear Bayesian ridge machine learning method.

Strikingly, We observe in Figure 2 that all the different machine learning methods correctly predicted the start-peak-end pollen seasonal cycles. However, we clearly observe that the four machine learning methods underestimated the pollen concentration during the unusually high pollen days and when the pollen abundance shows a high day to day variation. This is clearly observed when the daily pollen concentration is unusually high. For example, when the pollen concentration is nearly 1000 at the beginning of October 2013 and 2017, all the machine learning methods predicted about 250. While most of the methods tended to underestimate these unusually high pollen days, the deep neural networks machine learning method tries to fit well and even overestimates the pollen at times. For example, the deep neural network has overly estimated the pollen concentration on 8th October 2014. Generally unusually high pollen abundance measurements and large abrupt day to day variations are difficult to estimate by the machine learning methods. The underlying reason should be these abrupt changes in pollen concentrations are associated with atmospheric weather phenomenon that are not represented by any of the predictor parameters used in our machine learning models. Back trajectory analysis shows that these large day to day fluctuations in the pollen concentration are associated with long range transport from certain directions.

**Figure 3:**
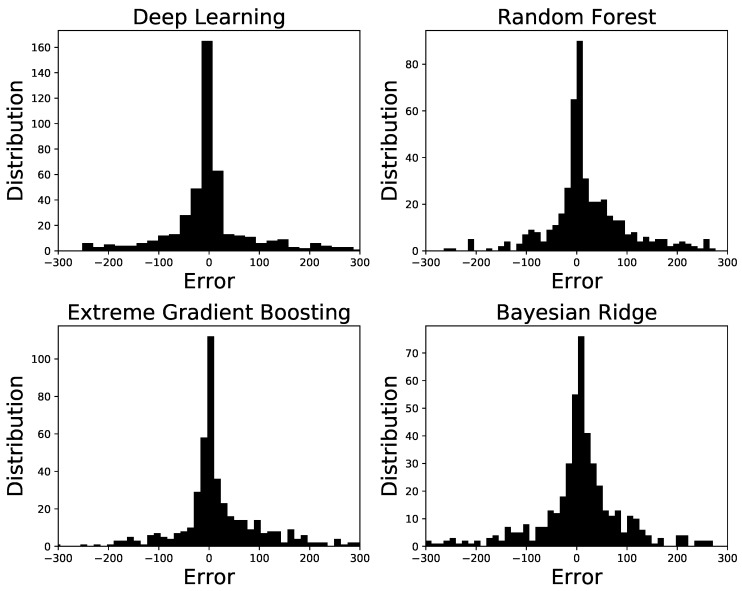
Histogram plots showing the distribution of the errors for the four machine learning methods.

Figure 3 shows the probability distributions of the estimated error between the predicted and the actual pollen (shown in Figure 2) for the four machine learning approaches used. The type of machine learning method for which the error is estimated is given at the top each panel. We observe that for all the four methods the error is symmetrically distributed about zero, showing the effective performance of the methods. However, we have error values as large as 200 to 300 with small number of occurrence as we go farther from zero on both sides. These poorer performances are associated with the days having an unusually high pollen concentrations (Figure 2).

**Figure 4:**
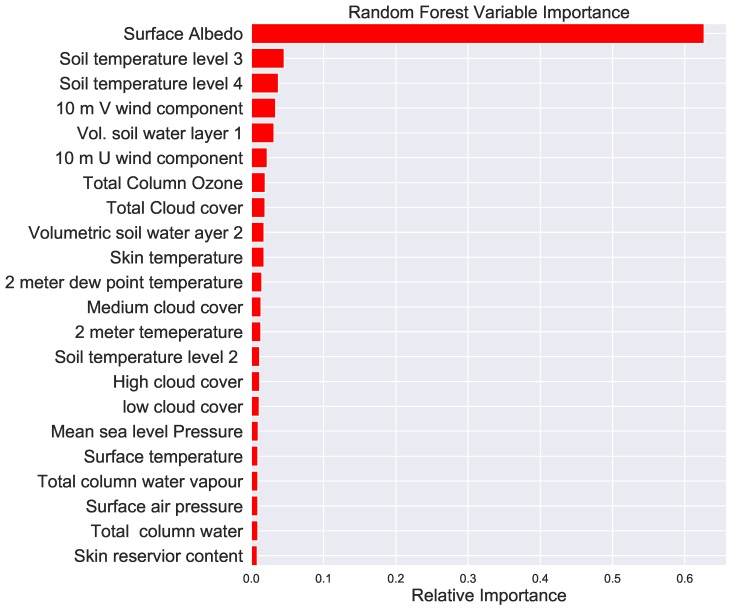
Variable importance of the parameters in decreasing order calculated using the random forest machine learning method before time lagging of the parameters is applied.

**Figure 5:**
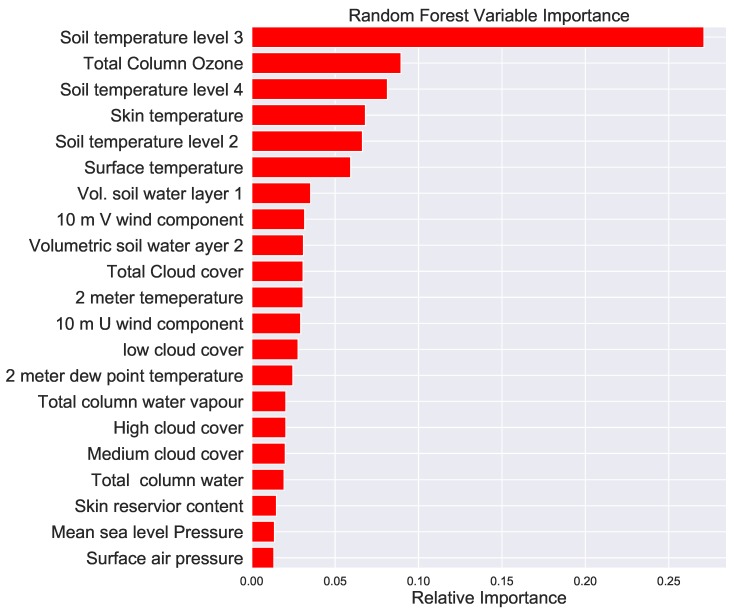
Variable importance of the parameters in decreasing order calculated using the random forest machine learning method before time lagging of the parameters is applied in the absence of the surface albedo.

The variable importance of of all the parameters in decreasing order is shown in Figure 4. The random forest machine learning method was used to estimate the variable importance. Note that the surface albedo which measures the fractional amount sunlight reflected by the Earth’s surface is the top predictor by a large amount. This was an interesting result as the amount Sun’s radiation reflected depends on the surface color of the Earth which in turn depends on the amount of vegetation cover producing allergic pollen. Note that soil temperature at various levels, the total column ozone, skin and 2 m temperatures were among the top predictors as shown by the variable importance estimation excluding the surface albedo (see Figure 5).

**Figure 6:**
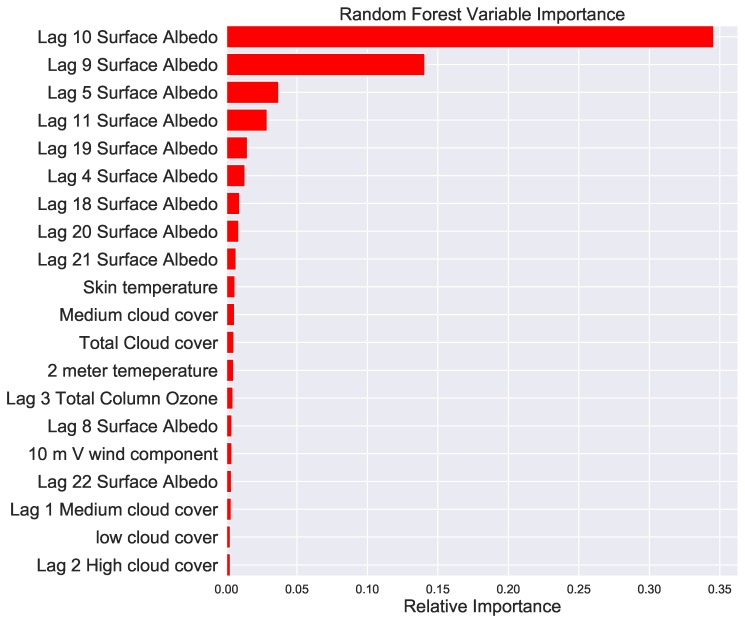
Variable importance of the parameters after lagging by 30 days estimated using the random forest machine learning method.

The result of the top 20 variable importance after lagging each parameter by between 1 and 30 days is shown in Figure 6. It is interesting to see that the surface albedo 9–10 days prior is the most important variable for predicting the present day’s pollen. Time lagging of the parameters slightly improves the Pearson correlation of the prediction despite the longer computation time.

It is observed in Figure 2 that high day to day variations in the pollen concentrations are challenging to predict for our machine learning algorithms. For example, we observe that most of our machine learning methods showed poor prediction when the pollen concentration is near or above 1000 for 1 October 2013 and 14–16 October 2017. Only deep neural networks exhibited comparatively better performance than the other methods during these high pollen episodes.

**Figure 7:**
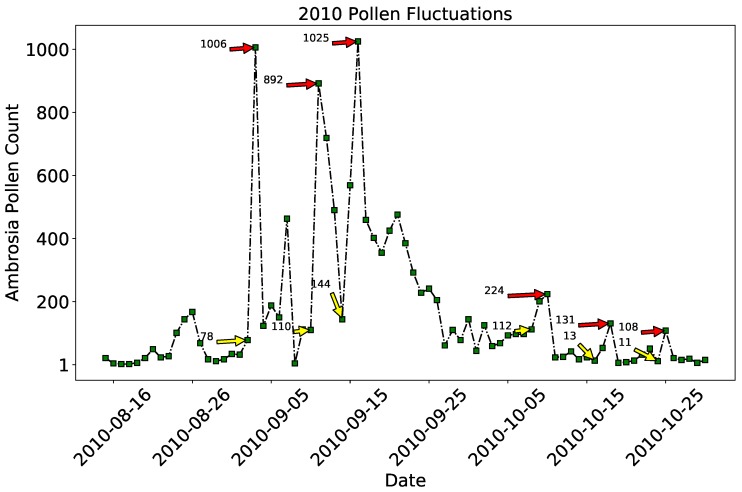
Large scale day to day variations of *Ambrosia* pollen concentration for the year 2010. These variations are challenging for forecasting the daily pollen abundance.

Examples of large scale day to day variations of the pollen abundance for 2010 are exhibited in Figure 7. The yellow arrows in Figure 7 show relatively low pollen abundance just one or two days before a large scale increase in the pollen indicated by the red arrows. The fluctuations are severe during the peak pollen days. For example, the pollen concentration recorded on 2nd October 2010 was just 78, and the following day (3rd October 2010) the pollen concentration sharply ascended to 1006. The 110 and 144 pollen concentrations on October 11 and 16 2010 increased to 892 and 1025, respectively. Similarly, during the pollen end days the abundance varies from 112 on 8th October, 13 on 16th October, 11 on 24th October to respectively 224 on 10th October, 131 on 18th October, 108 on 25th October. Understanding the causes of these large scale fluctuations in terms of atmospheric weather or land surface parameters will greatly help in forecasting the pollen abundance using machine learning.

**Figure 8:**
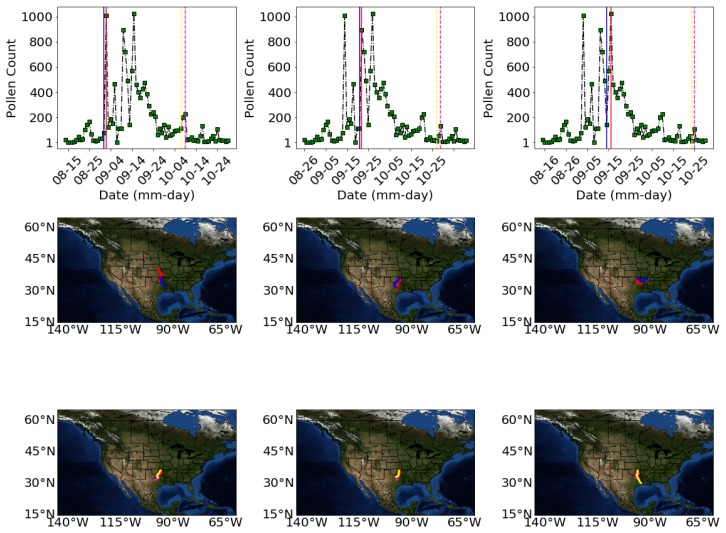
Result of back trajectory analysis showing sources of high magnitude *Ambrosia* pollen concentration in 2010. The top panel shows the days for which the trajectories are analyzed. The middle panel shows trajectories of the days of unusual increase in pollen concentration during peak season (red vertical lines) and the previous day (blue vertical lines). The bottom panel presents the unusual increase in the pollen concentration during the dying season (broken pink color) and the previous day (broken yellow color).

Figure 8 shows results of back trajectory analysis for the days of high pollen fluctuations and one or two days before these high pollen episodes to investigate the movement of the airborne pollen. The days we carried out the back trajectory analysis are shown by the arrows in Figure 7) for 2010. In Figure 8 the top panel shows the days for which the trajectories are analyzed. The middle panel shows trajectories of the days of unusual increase in pollen concentration during peak season (red vertical lines in the top panel) and the previous day (blue vertical lines in the top panel). The bottom panel presents the unusual increase in the pollen concentration during the end pollen season (broken pink color in the top panel) and the previous day (broken yellow color).

The back trajectories analyses indicate that large pollen fluctuations occur when the air parcels come mostly from the South on the same day or a day before. Most of these parcel appear to originate from North Texas. The shortness in length of the paths during these high pollen episodes indicate that the speed of the air parcels are slow.

## 4. Discussion

In this study we used deep neural networks, ensemble learners (random forest and extreme gradient boosting) and the Bayes ridge machine learning methods to forecast *Ambrosia* pollen at the University of Tulsa, Oklahoma (36.1511° N, 95.9446° W). We used atmospheric weather and land surface reanalyses data from the European Center for Medium-range weather Forecasting (ECMWF) [38]. In order to further investigate the relation between the previous days weather and land surface condition to the daily pollen abundance in the atmosphere, we also lagged all ECMWF parameters by 1–30 days back and used in our deep learning and machine learning algorithms.

Accurate forecasting of allergic pollen abundance in the atmosphere will help allergy sufferers in taking the necessary precaution. A reliable forecasting technique over highly populated urban areas are therefore valuable. Advanced machine learning and deep learning methods such as deep neutral networks, random forests, gradient boosting, support vector machines are the best candidates if we have large weather and land surface data spanning as large parameters as we can.

It is known that meteorological and land surface factors affect the production, dispersion and distribution of pollen in the atmosphere [45,46]. Various studies in the past have shown the relation between pollen distribution and meteorological factors using machine learning methods. For example, the relation between air temperature and pollen was shown by [26,47]. Refs. [20,48] showed the relation between pollen concentration, precipitation and atmospheric wind, respectively. However, a comprehensive model involving plant phytosociology, habitat, phenology and meteorological variables is needed to effectively estimate and forecast the atmospheric allergic pollen concentration in the ambient atmosphere over a large spatial area.

Previous studies have employed machine learning methods and meteorological variables to model allergic pollen abundance. For example, Csépe et al. [21] used multilayer perceptron neural networks and other tree algorithms to predict ragweed pollen abundance over Szeged (Hungary) and Lyon (France) using daily mean, maximum, minimum and range temperatures, daily mean wind speed, air pressure, total radiation and relative humidity and serial number of the day in the given year as predictor variables. Csépe et al. [21] found that the the daily total radiation (for Lyon) and the daily mean, max and range temperatures (for Szeged) are the most influential meteorological variables. Puc [26] used artificial neural networks and meteorological factors to estimate allergic pollen and showed that relative humidity and maximum temperature are the most important variables. Nowosad et al. [49] used different statistical methods including linear models, non-linear models such as neural networks and support vector machines and regression tree methods for different places and different pollen species. Nowosad et al. [49] used eleven meteorological variables including maximum, minimum, and mean temperatures, vapor pressure, wind speed precipitations, growing degree days, etc and found that growing degree days is the most important variable for all the three pollen species.

Identifying the most important predictor variables would help us to optimize our machine learning models to forecast pollen. In general, the list of important features varies on geographic location and the type of pollen species we are estimating.

In this research we used twenty-three predictor variables for our machine learning methods. The variables are time lagged by 1 to 30 days so that the total number of predictor variables becoming 31 × 23. We trained the machine learning before and after lagging the variables. The random forest was applied to estimate the variable importance. We found that the surface albedo, soil temperature and total column ozone are among the top most predictor variables. Time lagging of the variables shows that the surface albedo nine to 10 days prior are the most influential predictors.

However, pollen abundance is a complex function of weather, land surface and air transport and its modelling is challenging. The pollen distributions commonly exhibited large day to day variations which are hard to capture in machine learning models. Back-trajectory analysis showed that large day to day variations of pollen abundance are associated with directional air movement [34].

Future studies should include variables carrying information about the directional air parcel movement in order to of improve allergic pollen forecasting. Variables derived from back and forward trajectory analysis, Ref. [44] could improve in forecasting the challenging abrupt large scale day day variations in the pollen abundance.

## 5. Conclusions

In this study we used advanced machine learning (random forest, extreme gradient boosting and deep neural networks) to forecast the airborne abundance of *Ambrosia* pollen. For comparison we also used the linear Bayes ridge machine learning model. The *Ambrosia* pollen used to supervise the machine learning methods was measured at University of Tulsa, Oklahoma (1987–2017) using a Burkard trap. The environmental context used by the machine learning models to estimate the airborne pollen concentration were from ECMWF reanalysis data. This environmental context was also time lagged from between 1–30 days to examine the role of the recent historical environmental context on estimate the airborne pollen abundance.

Capability of forecasting allergic pollen would improve the life of allergic susceptible individuals by providing reliable information ahead of time. It will also provide useful information for health professionals who provide care for allergic people. This paper provides useful input in forecasting future pollen abundance by providing the relation between meteorological and land surface parameters with pollen.

We have found the surface albedo, soil temperature, total column ozone are the most important variables for the machine learning models. Time lagging the predictors has shown that the surface albedo 9–10 days a head is the most influential parameter.

The superior performance of the deep neural networks, random forest and extreme gradient boosting with correlation values 0.82, 0.81 and 0.81 respectively indicate the feasibility of advanced machine learning and deep learning methods to forecast allergic pollen a head of time. NOAA’s HYSPLIT back-trajectory analysis show that large scale day to day variations in the pollen concentration are when the air parcels come from a certain direction. Back-trajectory analysis performed at our pollen observation site indicate that high pollen episodes and large day to day fluctuations in pollen concentration are associated with the air mass coming from the Southern direction.

These large fluctuations in the pollen concentration are hard to model using advanced machine learning models. One reason can be the predictors in the training data may not contain a parameter representing these fluctuations. Including backward and forward trajectory data might improve the forecasting of pollen.

## Figures and Tables

**Table 1:** The European Centre for Medium-Range Weather Forecasts (ECMWF) predictor parameters used in our machine learning pollen estimates.

Parameter Name	Unit	Parameter Name	Unit
2 m temperature	K	2 m dew point temperature	K
Vol. soil water layer 1	m${}^{3}$ m${}^{-3}$	Soil temperature level 2	K
Vol. soil water layer 2	m${}^{3}$ m${}^{-3}$	Soil temperature level 3	K
Surface air pressure	Pa	Low cloud cover	0–1
Total column water	kg m${}^{-2}$	Medium cloud cover	0–1
Total column water vapour	kg m${}^{-2}$	High cloud cover	0–1
Surface temperature	K	Skin reservoir content	m
Mean sea level Pressure	Pa	Total column ozone	kg m${}^{-2}$
Total Cloud cover	0–1	Skin temperature	K
10 m U wind component	ms${}^{-1}$	Soil temperature level 4	K
10 m V wind component	ms${}^{-1}$	Surface Albedo	0–1
